# Utilizing Star Polycation Nanocarrier for the Delivery of miR-184 Agomir and Its Impact on the Life History Traits of the English Grain Aphid, *Sitobion avenae*

**DOI:** 10.3390/insects15060459

**Published:** 2024-06-19

**Authors:** Cong Zhang, Guohua Wei, Linyuan Wu, Yunhui Zhang, Xun Zhu, Austin Merchant, Xuguo Zhou, Xiangying Liu, Xiangrui Li

**Affiliations:** 1 College of Plant Protection, Hunan Agricultural University, Changsha 410128, China; m14735898652@163.com; 2 State Key Laboratory for Biology of Plant Diseases and Insect Pests, Institute of Plant Protection, Chinese Academy of Agricultural Sciences, Beijing 100193, China; w2950390082@163.com (G.W.); wulinyuan0525@163.com (L.W.); yhzhang@ippcaas.cn (Y.Z.); zhuxun@caas.cn (X.Z.); 3 Department of Entomology, Martin-Gatton College of Agriculture, Food and Environment, University of Kentucky, Lexington, KY 40546, USA; ajme232@uky.edu; 4 Department of Entomology, School of Integrative Biology, College of Liberal Arts & Sciences, University of Illinois Urbana-Champaign, Urbana, IL 61801, USA; xgzhou@illinois.edu

**Keywords:** genetics-based pesticide, microRNA, star polycation, two-sex life table, growth and development, interference agent

## Abstract

**Simple Summary:**

This study investigates the effectiveness of a novel genetics-based biopesticide, miR-184 agomir, against the English grain aphid, *Sitobion avenae*, a major wheat pest. miR-184 agomir interference significantly reduced aphid survival rates, particularly during their early developmental stages. Life table analysis demonstrated that the application of miR-184 agomir resulted in adverse effects on multiple vital parameters essential for the expansion of the aphid population. Population projection models predicted a substantial decline in the aphid population size at 60 days post-treatment. These findings underscore the potential of genetics-based biopesticides in the effective management of grain aphid populations, thereby contributing to wheat crop protection and environmental sustainability.

**Abstract:**

The investigation of genetics-based biopesticides has become a central focus in pesticide studies due to their inherent advantages, including species specificity, environmental safety, and a wide range of target genes. In this study, a mixture of miR-184 agomir and nanomaterial star polycation (SPc) was used to treat the nymphs of the English grain aphid, *Sitobion avenae* (F.). The life parameters of the aphids at various developmental stages were analyzed using an age–stage two-sex life table to assess the effect of miR-184 agomir on the experimental population. The results indicated that miR-184 agomir had a significant negative effect on four key life parameters, including the intrinsic rate of increase, the finite rate of increase, the net rate of increase, and the mean generation time. The population prediction revealed a substantial reduction (91.81% and 95.88%) in the population size of *S. avenae* at 60 d after treatment with miR-184 agomir, compared to the control groups. Our findings suggest that the miR-184 agomir has the potential to reduce the survival rate and mean longevity of *S. avenae*, highlighting its potential as a promising candidate for the development of an effective genetics-based biopesticide.

## 1. Introduction

Wheat, *Triticum aestivum* (L.), is a staple crop of paramount importance worldwide. However, it faces significant threats from pests such as the English grain aphid, *Sitobion avenae* Fabricius (Aphididae, Hemiptera), which severely impacts yields through sap extraction from the phloem and transmission of barley yellow dwarf virus (BYDV). BYDV causes dwarfing, stunting, and chlorosis in wheat, leading to further yield reductions [1,2,3,4]. Traditionally, control measures for *S. avenae* have relied heavily on chemical pesticides. Unfortunately, the extensive and often indiscriminate utilization of these chemicals has led to increasing resistance in aphid populations against multiple chemical pesticides [5,6,7], significantly compromising the efficacy of current management strategies. Consequently, there is an urgent need for innovative, environmentally benign, and effective pesticide solutions to address these challenges.

Pest control methods based on mechanisms that modify gene expression have shown great potential due to their simplicity, high efficiency, and potential specificity. RNA interference (RNAi) is among the most comprehensively studied of these mechanisms and shows great potential in the development of novel biopesticides [8]. RNAi leads to gene knockdown through the use of double-stranded RNA (dsRNA) molecules [9]. Although dsRNA is generally stable in Coleoptera, resulting in a high level of RNAi efficiency, dsRNA in Hemiptera and Lepidoptera is prone to degradation, limiting the effectiveness of RNAi in these types of insects [10]. One alternative lies in the use of microRNA (miRNA) to suppress the target genes. Recent research underscores the critical role of miRNA in regulating the biological functions of insects. Insects resistant to dsRNA-induced RNAi might be responsive to miRNA-mediated methods of gene knockdown [11,12]. For instance, transgenic rice expressing an endogenous insect miRNA, csu-novel-260, has shown resistance to the striped stem borer, *Chilo suppressalis* (Crambidae, Lepidoptera) under field conditions [13]. miRNA has also been shown to play a pivotal role in the metamorphic development of the cotton bollworm, *Helicoverpa armigera* (Noctuidae, Lepidoptera), and the aberrant expression of specific miRNAs can cause molting defects [14,15]. These and other findings demonstrate the potential of miRNA as a mechanism for gene knockdown in the development of novel biopesticides.

miR-184 is a conserved miRNA in insects that is known to influence an increasingly larger number of biological processes. In *Drosophila* (Drosophilidae, Diptera), it is expressed in the germline and in eggs, and plays roles during oogenesis and embryogenesis [16], while in the migratory locust, *Locusta migratoria* (Acrididae, Orthoptera), it regulates the molting process by influencing the expression of the *LmCYP303A1* gene, a crucial component in the insect life cycle [17]. In the pea aphid, *Acyrthosiphon pisum* (Aphididae, Hemiptera)*,* miR-184 negatively regulates the JNK (Jun N-terminal kinase) signaling pathway, which is involved in the immune response [18]. Moreover, miR-184 impacts the plant virus–host interactions by regulating the dynamics between rice black streak dwarf virus (RBSDV) and its insect vector, the planthopper *Laodelphax striatellus* (Delphacidae, Hemiptera), offering insights into pest-mediated virus transmission [19].

In a previous study conducted by our team, it was found that feeding *S. avenae* with a miR-184 agomir induced mortality at rates of up to 69% [20]. However, the feeding method previously used did not allow for the quantification of the amount of miR-184 agomir ingested. To address this limitation, we developed a delivery system using a previously described star polycation (SPc) as a carrier, which is an efficient and economical gene vector for pest management. SPc, sized at 100.5 nm and with its core structure featuring four arms each containing a compact tertiary amine structure, exhibits superior gene transfection capabilities [21]. Notably, the SPc-mediated delivery of precursor miRNA has been shown to effectively the increase levels of mature miRNA, consequently inhibiting the expression of target genes [22]. We evaluated its effectiveness in controlling *S. avenae* and studied its impact on the population parameters using life table analysis. These findings offer valuable insights for the potential application of miR-184 agomir as a biopesticide in managing *S. avenae* populations.

## 2. Materials and Methods

### 2.1. Insects

*Sitobion. avenae* were collected from a wheat field at the Scientific Observatory of Crop Pests (Ministry of Agriculture and Rural Affairs) in Langfang, Hebei Province, China (39°30′42″ N, 116°36′7″ E) in 2012, and reared indoors for several generations. For the experiment, disposable 9 cm diameter Petri dishes lined with qualitative filter paper were prepared. Each dish contained several 1.5 mL centrifuge tubes with 3~4 fresh hydroponically grown wheat seedlings (Zhong Mai 175, an aphid-susceptible variety). The dishes were routinely sprayed with fresh water, and the wheat seedlings were replaced every three days. Healthy adult aphids were selected from the rearing cage and attached to the wheat seedlings. After adult aphids produced nymphs, individual nymphs were transferred to new Petri dishes and reared for three consecutive generations before being used in experiments. The rearing conditions included a temperature maintained at 20 ± 1 °C, a photoperiod of 16:8 (L:D) hours, and relative humidity between 50% and 70%.

### 2.2. Test Agents

The miR-184 agomir and negative control agomir (NC agomir) (sequence in Table S1, synthesized by Shanghai Genepharma Pharmaceutical Technology Co., Ltd., Shanghai, China) were mixed at a 1:1 mass ratio with the nanomaterial SPc (provided by Dr. Jie Shen’s team at China Agricultural University). To this mixture a 0.2% additive, alkyl polyglucoside (APG) (Shanghai Yuanye Biotechnology Co., Ltd., Shanghai, China), was added to formulate a complex solution. To determine the optimal dose of miR-184 agomir for *S. avenae* treatment, a preliminary experiment was conducted. First-instar nymphs were treated with 0.2 μL of miR-184 agomir at various concentrations (200–700 nmol/L). Mortality rates peaked at 400 nmol/L and stabilized thereafter (Figure S1). Consequently, 0.2 μL of 400 nmol/L was chosen as the optimal dose.

### 2.3. Experimental Methods for Life Tables

After three generations of rearing, first-instar nymphs were individually placed in disposable Petri dishes, each containing a single wheat seedling. Each nymph was assigned to one of three groups: the treatment group, which received miR-184 agomir (400 nmol/L), SPc, and additives; the clear water control, which received only ddH_2_O (double distilled water); and the SPc control, which received NC agomir, SPc, and additives. Each group consisted of 150 aphids. The solution corresponding to the individual’s group was administered to the dorsal plate of the nymph using a microinjector (Hamilton, Hamburg, Germany), with each nymph receiving a 0.2 µL dose. Treatments were applied at 24 h over two consecutive days. Aphid growth, development, and mortality were monitored at 24 h intervals post-inoculation. The shed skins of molting aphids and dead aphids were removed daily. The number of offspring produced by each adult aphid was recorded daily until the adult’s death.

### 2.4. Data Analysis

Raw data were analyzed with an age-stage, two-sex life table [23] using the TWOSEX-MSChart software (Version 2023.05.07) [24]. This theory and its software consider the unique aspect of solitary reproduction in aphids. Population parameters such as age-stage specific survival rate (*S_xj_*), age-stage specific fecundity (*f_xj_*), age-specific survival rate (*l_x_*), and age-specific fecundity (*m_x_*), were calculated as follows, in which “*x*” represents age and “*j*” represents stage [25]:

(1) $$S_{xj}=\frac{n_{xj}}{n_{0,1}}$$

(2) $$f_{xj}=\frac{f_{x,total}}{n_{xj}}$$

(3) $$l_{x}=\frac{\sum_{j=1}^{k}n_{xj}}{n_{0,1}}=\sum_{j=1}^{k}s_{xj}$$

(4) $$m_{x}=\frac{\sum_{j=1}^{k}s_{xj}f_{xj}}{\sum_{j=1}^{k}s_{xj}}=\frac{\sum_{j=1}^{k}s_{xj}f_{xj}}{l_{x}}$$

The population parameters were analyzed using the following equations:

For a traditional female age-specific life table, the net reproductive rate (*R*_0_) of a female population is defined as:

(5) $$R_{0}=\sum_{x=0}^{\infty }l_{x}m_{x}$$

The intrinsic rate of increase (*r*) is calculated using the iterative bisection method and the Euler–Lotka equation, starting from the birth of the aphid:

(6) $$\sum_{x=0}^{\infty }e^{-r\left(x+1\right)}l_{x}m_{x}=1$$

Finite rate (*λ*):

(7) $$\lambda =e^{r}$$

Mean generation time (*T*):

(8) $$T=\frac{ln{(R}_{0})}{r}$$

The means and standard errors of population parameters were estimated using the paired bootstrap test [26,27], with the bootstrap set to 100,000 replicates to minimize statistical errors [28,29]. To assess statistical differences among the treatment groups, a paired bootstrap test (B = 100,000) was employed. This test evaluates the differences based on the percentile and the 95% confidence interval (CI) of the normalized distribution of differences [30,31].

### 2.5. Population Projections

Population dynamics for 150 F1 generations of wheat aphids under each treatment condition were projected using TIMING-MSChart software (Version 2023.06.26) [32,33], based on life table calculations. This analysis excluded external factors such as disease, predation, and parasitism. All the graphical representations of curves were generated using GraphPad Prism 8.3, ensuring the high-quality visualizations of the data.

## 3. Results

### 3.1. Effect of miR-184 Agomir Treatment on Growth and Development of S. avenae

The *S. avenae* treated with miR-184 agomir successfully completed their life cycle, with total longevity showing no significant difference compared to the two control groups. However, the developmental duration at the second and third instar nymph stages in the miR-184 agomir treatment group was significantly longer than in the water control group, extended by 0.56 and 0.23 days, respectively (*p* < 0.05). Overall, the duration of the nymphal stage for the miR-184 agomir treatment group was prolonged by an average of 0.70 days compared to the water control (*p* < 0.05). No significant difference was observed in the duration of the adult stage between *S. avenae* treated with miR-184 agomir and the control groups (Table 1).

### 3.2. Effect of miR-184 Agomir Treatment on Population Parameters of S. avenae Adult Aphids

The mean longevity of *S. avenae* treated with miR-184 agomir was 12.9 days, which was significantly shorter than that observed in the SPc (17.31 days) and water (18.61 days) control groups. The survival rate for pre-adults in the miR-184 agomir treatment group was 53%, significantly lower than in the SPc (84%) and water (89%) control groups. While there was no significant difference in the duration of the oviposition period between the miR-184 agomir-treated group and the control groups, a significant reduction in fecundity was noted. Specifically, the average number of offspring produced per female in the miR-184 agomir group was 3.36 and 4.82 fewer than in the SPc and water control groups, respectively (*p* < 0.05) (Table 2).

### 3.3. Effect of miR-184 Agomir Treatment on Survival Rate and Survival Time of S. avenae

**Figure 1:**
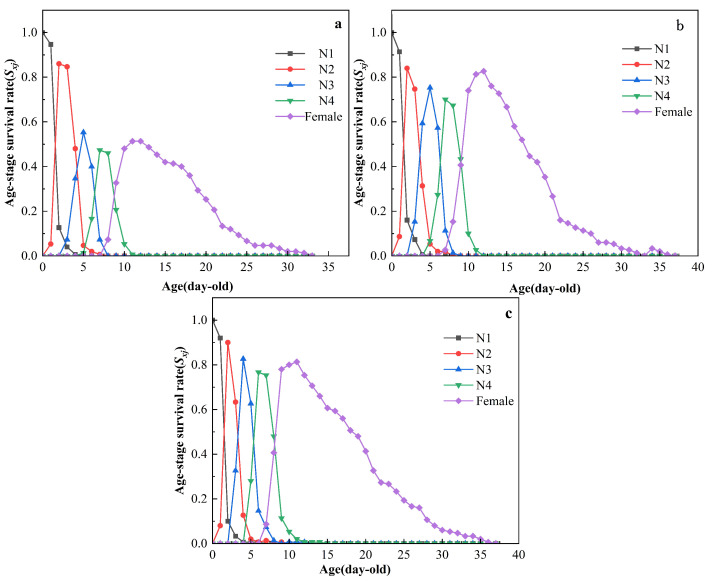
Age-stage-specific survival rate (*S_xj_*) of *S. avenae* after treatment with (**a**): miR-184 agomir; (**b**): SPc; (**c**): water; N1~N4: 1st–4th instar nymph; Female: Female adult aphids.

The age-stage specific survival rate (*S_xj_*) curves illustrate the survival rate of *S. avenae* from the initial nymph stage to age *x* and stage *j* (Figure 1). The curves overlap, indicating temporal overlap between different developmental stages. The survival rate for first instar miR-184 agomir-treated nymphs was 93.33%, similar to the controls (SPc: 94%, water: 97.33%). However, survival rates for second and third instar miR-184 agomir-treated nymphs were significantly lower at 65.71% and 88.04%, respectively, compared to the SPc control (second instar: 91.48%, third instar: 98.45%) and the water control (second instar: 95.89%, third instar: 97.86%) (*p* < 0.05). The survival rate for 4th instar miR-184 agomir-treated nymphs was 98.77%, which was comparable to the SPc control (99.21%) and water control (97.81%) (Table S2). The maximum lifespan of adult aphids treated with miR-184 agomir was 33 days, while that of the SPc and water control groups was 37 days.

**Figure 2:**
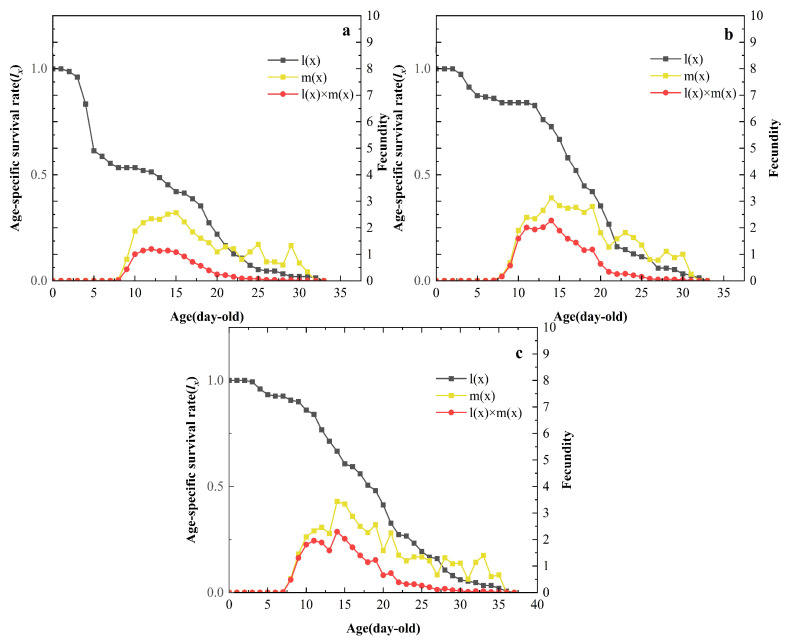
Age-specific survival rates (*l_x_*), age-specific fecundity (*m_x_*), and age-specific net maternity (*l_x_m_x_*) of *S. avenae* after treatment with (**a**): miR-184 agomir; (**b**): SPc; (**c**): water.

The age-specific survival (*l_x_*) curves for the three treatments showed a steeper decline between 15 and 20 days, indicating a period of higher mortality during the late adult stage (Figure 2). The miR-184 agomir-treated group exhibited higher mortality in the second to third nymphal instars during the pre-growth and developmental period (2–5 days). Age-specific fecundity (*m_x_*) trends increased and then decreased, with peak fecundity occurring at 14–16 days. The maximum fecundity for the miR-184 agomir-treated group was 2.57 offspring per day, lower than the 3.12 offspring per day observed in the SPc control and the 3.44 offspring per day observed in the water control. The age-specific net fecundity curve (*l_x_m_x_*) for the miR-184 agomir treatment was significantly lower, with a maximum of 1.13, compared to 2.27 for the SPc control and 2.29 for the water control.

### 3.4. Effect of miR-184 Agomir Treatment on Life Table Parameters in S. avenae Populations

The life table parameters for miR-184 agomir-treated *S. avenae* populations showed significant differences compared to the controls. The intrinsic rate of increase (*r*) was reduced by 0.0315 and 0.0413, the finite rate of increase (*λ*) decreased by 0.0381 and 0.0503, and the net reproductive rate (*R*_0_) decreased by 7.01 and 8.55 compared to the SPc and water control groups, respectively (*p* < 0.05) (Table 3). The mean generation time (*T*) was significantly prolonged by 0.61 days compared to the water control (*p* < 0.05).

### 3.5. Prediction of S. avenae Population Followed by miR-184 Agomir Treatment

**Figure 3:**
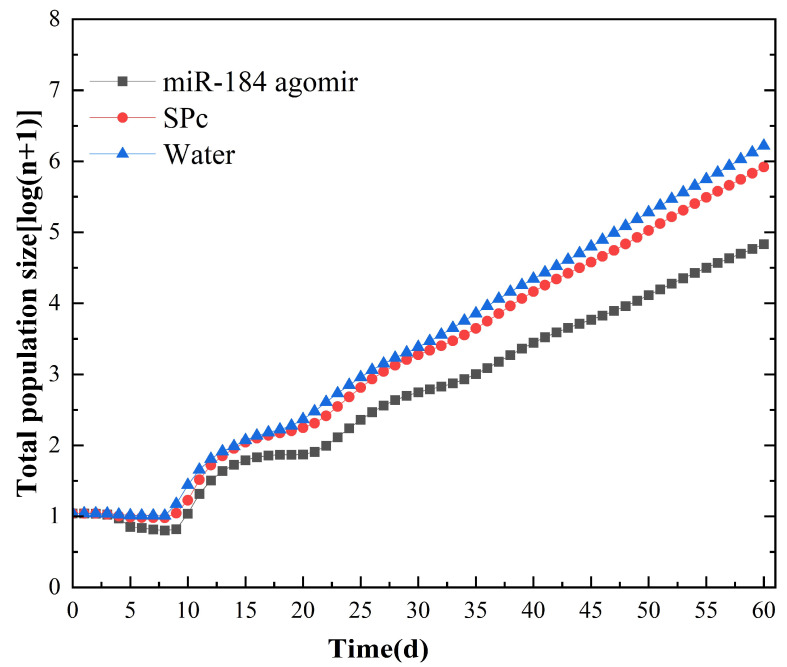
Comparison of predicted population dynamics of *S. avenae* after miR-184 agomir treatment.

Population dynamics predictions for *S. avenae* were made using data from the age-stage, two-sex life table. After 40 days, the aphid population exhibited linear growth with a rate corresponding to the logarithm of *λ* (log(*λ*)), nearly reaching a stable age-stage distribution (Figure 3). Predicted population sizes at 60 days post-treatment were markedly different among the groups: 68,000 for miR-184 agomir-treated aphids, compared to the NC SPc, the population decreased by 91.81% (830,000), and compared to the NC water, the population decreased by 95.88% (1,650,000).

## 4. Discussion

Biopesticides that manipulate the gene expression of their targets offer advantages over traditional chemical pesticides, including species specificity, environmental safety, and targeting a wide range of genes [34,35,36]. Although RNAi shows high potential in this regard, its effectiveness varies across insect orders because the dsRNAs introduced into insects via injection or feeding are susceptible to degradation by nucleases present in body fluids [37]. Therefore, in insects that exhibit low RNAi efficiency, genetic control would be best achieved through alternative methods. Here, we observed the effects of miRNA treatment on the English grain aphid, *S. avenae*, an important hemipteran pest, by exposing them to a miR-184 agomir combined with the nanomaterial SPc as a carrier, which is an efficient and low-cost nanocarrier that protects RNA from degradation, facilitating its effective uptake [21].

The survival rate of *S. avenae* pre-adults treated with miR-184 agomir was significantly lower than that of SPc and water control groups, particularly during the second and third instar nymph stages. This reduction in survival rates confirms the potential of miR-184 agomir as a viable candidate for genetics-based biopesticide development [20]. While the specific molecular mechanisms through which miR-184 agomir induces mortality in *S. avenae* remain to be fully elucidated, existing research suggests that miR-184 could disrupt essential physiological processes. For example, Ma et al. [22] demonstrated that the injection of miR-184 agomir led to a significantly reduced expression of JNK mRNA in the pea aphid *A. pisum* (Aphididae, Hemiptera), along with decreases in phenoloxidase (PO) activity, hydrogen peroxide concentration, and hemocyte phagocytosis in the JNK signaling pathway, which were associated with increased mortality rates. Interestingly, in our study, the second and third instars of *S. avenae* showed high mortality rates after treatment with miR-184 agomir. Similarly, in the migratory locust, *L. migratoria* (Acrididae, Orthoptera), the injection of miR-184 agomir resulted in the downregulation of the cytochrome P450 monooxygenase gene *LmCYP303A1*, causing abnormal molting and high mortality during the second and third instars [17].

miR-184 agomir treatment caused significant mortality among the second and third instar nymphs of *S. avenae* and subtly affected their developmental durations. Specifically, the developmental duration of *S. avenae* nymphs was significantly prolonged in the miR-184 agomir-treated group compared to the water control group. This observation aligns with findings in *Drosophila melanogaster* (Drosophilidae, Diptera), where miR-184 plays a role during early developmental stages. The low expression of miR-184 in fruit flies results in severe defects during oogenesis and early embryogenesis, ultimately leading to a loss of oviposition capability [16]. This parallels our findings and underscores the pivotal role of miR-184 in developmental processes. Moreover, we observed a significant reduction in the mean longevity of *S. avenae* by approximately 5 days in the miR-184 agomir-treated group in comparison to the controls. Prior studies on *Drosophila* have demonstrated that the overexpression of miR-184 can decrease longevity by 10 days, irrespective of food supply conditions [38]. This evidence suggests a link between miR-184 and insect lifespan, indicating that miR-184 could influence longevity, though the specific underlying mechanisms warrant further investigation.

Following the treatment with miR-184 agomir, the experimental population of *S. avenae* exhibited significant reductions in life table parameters, including the intrinsic rate of increase^®^, finite rate of increase (*λ*), net reproductive rate (*R*_0_), and mean generation time (*T*). These results indicate that miR-184 agomir treatment has a substantial inhibitory effect on the population growth of *S. avenae*. Population prediction models further showed a significant decrease in the population size of *S. avenae* within 60 days post-treatment, mirroring the effects observed with sub-lethal concentrations of imidacloprid [39]. This similarity suggests that miR-184 agomir can inhibit the growth of *S. avenae* populations in a manner analogous to traditional chemical pesticides. Given the increasing number of reports of pest resistance associated with the use of chemical pesticides [5,6,7], genetics-based biopesticides have attracted significant attention for their theoretical specificity and environmental friendliness. Our findings in this study affirm the potential of miRNA-184 as a candidate novel biopesticide for effectively controlling *S. avenae*.

In our study, we introduced SPc as a delivery vector, which successfully facilitated the delivery of miR-184 agomir. This strategic approach resulted in significant mortality among *S. avenae*, effectively curbing the growth of the experimental population. Compared to previous methodologies, such as feeding methods, which suffered from limitations like quantifying miRNA, susceptibility to degradation, and inadequate ingestion, the SPc-based approach significantly improves miRNA stability and delivery efficiency. This not only enables effective gene interference at lower doses but also allows for the more precise quantification and control of miRNA. Furthermore, the potential for deploying spray methods for large-scale control applications, combined with the low cost of SPc, significantly enhances the possible practical utility of this technology [40], marking a substantial advancement in managing agricultural pests through genetic means. Genetics-based biopesticides offer numerous advantages over traditional chemical pesticides, confronting the challenges concerning resistance and biosafety. The capability of miRNA to simultaneously target multiple genes plays a crucial role in delaying the development of resistance against genetics-based biopesticides. This effect could be further enhanced by combining these biopesticides with conventional chemical pesticides to extend their efficacy, offering a comprehensive and sustainable solution for pest management [41].

Regarding biosafety, studies indicate that the ubiquitous presence of nucleases in vertebrates capable of degrading RNA ensures that RNAi-related RNA is rapidly decomposed upon entering the digestive system, posing no threat to vertebrates [42]. The biosafety of RNAi for beneficial organisms, such as bees, has also been investigated. In studies where dsRNA targeting genes lethal to the Western corn rootworm was applied to bees, no adverse effects were observed on adult or larval bees, even at high dosages [42]. However, there has not been an assessment of the biosafety of miRNA on beneficial organisms. Considering the broad spectrum of miRNA regulatory characteristics, the control strategy we propose still needs further investigation in future research to assess its biosafety impacts on non-target species.

This study employed miR-184 agomir to interfere with the English grain aphid, *S. avenae*, suggesting that miR-184 may play an essential role in the pest’s growth and development. However, the precise molecular mechanisms through which miR-184 influences *S. avenae*’s growth and development remain to be determined. Future research should focus on elucidating miR-184’s regulatory mechanisms to enhance our understanding of its potential in genetics-based pest control strategies. In this research, we employed a nanomaterial, SPc, which was blended with miR-184 agomir to formulate an interference agent that, upon application to *S. avenae*, demonstrated effective disruption. Given miR-184’s significant role during the growth and development of various insects, the miR-184 agomir disruptor chosen for our study might also possess lethal effects on other pest species. Biopesticides developed based on miR-184 could potentially exhibit broad-spectrum insecticidal properties for further pest management. Despite the challenge of high synthesis costs currently hindering the widespread application of miR-184 agomir for *S. avenae* control, ongoing scientific advancements suggest that its utilization as a biopesticide may soon become viable.

## Figures and Tables

**Table 1:** The developmental duration of *S. avenae* after miR-184 agomir treatment.

Development Duration	miR-184 Agomir	NC SPc	NC Water
1st instar nymph	2.06 ± 0.04 a	2.06 ± 0.05 a	2.00 ± 0.03 a
2nd instar nymph	2.34 ± 0.06 a	2.14 ± 0.06 ab	1.78 ± 0.04 b
3rd instar nymph	2.4 ± 0.08 a	2.56 ± 0.06 a	2.17 ± 0.05 b
4th instar nymph	2.58 ± 0.07 a	2.69 ± 0.05 a	2.73 ± 0.05 a
Pre-adult	9.36 ± 0.10 a	9.45 ± 0.09 a	8.66 ± 0.09 b
Adult longevity	10.58 ± 0.55 a	10.24 ± 0.43 a	11.45 ± 0.58 a
Total longevity	19.94 ± 0.54 a	19.69 ± 0.44 a	20.11 ± 0.58 a

Note: Data in the table are represented as mean ± SE estimated with bootstrapping (100,000). All values are given in days. Different letters in the same row indicate significantly difference (*p* < 0.05, paired bootstrap test).

**Table 2:** The population parameters of *S. avenae* after miR-184 agomir treatment.

Parameters	miR-184 Agomir	NC SPc	NC Water
Mean longevity (d)	12.9 ± 0.68 b	17.31 ± 0.58 a	18.61 ± 0.63 a
Fecundity	20.1 ± 1.27 b	23.76 ± 1.15 a	24.92 ± 1.43 a
Oviposition period (d)	9.00 ± 0.75 a	9.00 ± 0.48 a	9.00 ± 0.60 a
Pre-adult survival	0.53 ± 0.04 b	0.84 ± 0.03 a	0.89 ± 0.03 a

Note: Data in the table are represented as mean ± SE estimated with bootstrapping (100,000). Different letters in the same row indicate significant differences (*p* < 0.05, paired bootstrap test).

**Table 3:** Population life table parameters of *S. avenae* after miR-184 agomir.

Parameters	miR-184 Agomir	NC SPc	NC Water
Intrinsic rate of increase *r*	0.1754 ± 0.0067 b	0.2069 ± 0.0040 a	0.2167 ± 0.0044 a
Finite rate of increase *λ*	1.1917 ± 0.0079 b	1.2298 ± 0.0049 a	1.2420 ± 0.0054 a
Net reproductive rate *R*_0_	13.71 ± 1.35 b	20.72 ± 1.30 a	22.26 ± 1.43 a
Mean generation time *T* (d)	14.93 ± 0.17 a	14.65 ± 0.17 ab	14.32 ± 0.17 b

Note: Data in the table are represented as the mean ± SE estimated with bootstrapping (100,000). Different letters in the same row indicate significant differences (*p* < 0.05, paired bootstrap test).

## Data Availability

The data presented in this study are available in the article.

## Supplementary Materials

The following supporting information can be downloaded at: https://www.mdpi.com/article/10.3390/insects15060459/s1, Figure S1: The impact of the different concentrations of miR-184 agomir on the mortality of *S. avenae*; Table S1: Sequence information of miRNA agomir; Table S2: Age-stage-specific survival rate (*S_xj_*) of *S. avenae* after miR-184 agomir treatment.

## References

[1] Hoffman T.K., Kolb F.L. (1998). Effects of barley yellow dwarf virus on yield and yield components of drilled winter wheat. Plant Dis..

[2] Larsson H. (2005). A crop loss model and economic thresholds for the grain aphid, *Sitobion avenae* (F.), in winter wheat in southern Sweden. Crop Prot..

[3] Li D.D., Su D., Tong Z.Q., Zhang C., Zhang G.S., Zhao H.Y., Hu Z.Q. (2019). Virus-Dependent and -Independent Responses of Sitobion avenae (Homoptera: Aphididae) Feeding on Wheat Infected by Transmitted and Nontransmitted Viruses at Transcriptomic Level. J. Econ. Entomol..

[4] Van Emden H.F., Harrington R. (2007). Aphids as Crop Pests.

[5] Fontaine S., Caddoux L., Barres B. (2023). First report of the kdr pyrethroid resistance mutation in a French population of the English grain aphid, Sitobion avenae. Crop Prot..

[6] Gong P.P., Li X.N., Gao H.F., Wang C., Li M.Y., Zhang Y.H., Li X.R., Liu E.L., Zhu X. (2021). Field evolved resistance to pyrethroids, neonicotinoids, organophosphates and macrolides in Rhopalosiphum padi (Linnaeus) and Sitobion avenae (Fabricius) from China. Chemosphere.

[7] Liu A., Ru T., Wang X., Li S. (2001). Sensitivity Determination of Two Species of Aphids to Insecticides. Plant Prot..

[8] Yan S., Ren B., Zeng B., Shen J. (2020). Improving RNAi efficiency for pest control in crop species. Biotechniques.

[9] Fire A., Xu S., Montgomery M.K., Kostas S.A., Driver S.E., Mello C.C. (1998). Potent and specific genetic interference by double-stranded RNA in Caenorhabditis elegans. Nature.

[10] Singh I.K., Singh S., Mogilicherla K., Shukla J.N., Palli S.R. (2017). Comparative analysis of double-stranded RNA degradation and processing in insects. Sci. Rep..

[11] Behura S.K. (2007). Insect microRNAs: Structure, function and evolution. Insect Biochem. Mol. Biol..

[12] Zhang Q., Dou W., Taning C.N.T., Smagghe G., Wang J.J. (2021). Regulatory roles of microRNAs in insect pests: Prospective targets for insect pest control. Curr. Opin. Biotechnol..

[13] Zheng X.X., Weng Z.J., Li H., Kong Z.C., Zhou Z.H., Li F., Ma W.H., Lin Y.J., Chen H. (2021). Transgenic rice overexpressing insect endogenous microRNA csu-novel-260 is resistant to striped stem borer under field conditions. Plant Biotechnol. J..

[14] Shen Z.J., Liu Y.J., Zhu F., Cai L.M., Liu X.M., Tian Z.Q., Cheng J., Li Z., Liu X.X. (2020). MicroRNA-277 regulates dopa decarboxylase to control larval-pupal and pupal-adult metamorphosis of Helicoverpa armigera. Insect Biochem. Mol. Biol..

[15] Shen Z.J., Zhu F., Liu Y.J., Li Z., Moural T.W., Liu X.M., Liu X. (2022). MicroRNAs miR-14 and miR-2766 regulate tyrosine hydroxylase to control larval-pupal metamorphosis in Helicoverpa armigera. Pest Manag. Sci..

[16] Iovino N., Pane A., Gaul U. (2009). miR-184 Has Multiple Roles in Drosophila Female Germline Development. Dev. Cell.

[17] Wang Y.L., Wu L.X., Li H.Y., Wen X.Q., Ma E.B., Zhu K.Y., Zhang J.Z. (2021). The microRNA miR-184 regulates the CYP303A1 transcript level to control molting of Locusta migratoria. Insect Sci..

[18] Ma L., Liu L., Zhao Y., Yang L., Chen C., Li Z., Lu Z. (2020). JNK pathway plays a key role in the immune system of the pea aphid and is regulated by microRNA-184. PLoS Pathog.

[19] Wu W., Wang M., Deng Z., Xi M., Dong Y., Wang H., Zhang J., Wang C., Zhou Y., Xu Q. (2023). miR-184-3p promotes rice black-streaked dwarf virus infection by suppressing Ken in Laodelphax striatellus (Fallén). Pest Manag Sci.

[20] Li X.R., Zhang F.M., Coates B., Wei C.P., Zhu X., Zhang Y.H., Zhou X.G. (2022). Temporal analysis of microRNAs associated with wing development in the English grain aphid, Sitobion avenae (F.) (Homoptera: Aphidiae). Insect Biochem. Mol. Biol..

[21] Li J., Qian J., Xu Y., Yan S., Shen J., Yin M. (2019). A Facile-Synthesized Star Polycation Constructed as a Highly Efficient Gene Vector in Pest Management. ACS Sustain. Chem. Eng..

[22] Yang J., Yan S., Xie S., Yin M., Shen J., Li Z., Zhou Y., Duan L. (2022). Construction and application of star polycation nanocarrier-based microRNA delivery system in Arabidopsis and maize. J. Nanobiotechnology.

[23] Chi H., Su H.Y. (2006). Age-stage, two-sex life tables of Aphidius gifuensis (Ashmead) (Hymenoptera: Braconidae) and its host Myzus persicae (Sulzer) (Homoptera: Aphididae) with mathematical proof of the relationship between female fecundity and the net reproductive rate. Environ. Entomol..

[24] Chi H., Güncan A., Kavousi A., Gharakhani G., Atlihan R., Özgökçe M.S., Shirazi J., Amir-Maafi M., Maroufpoor M., Roya T. (2022). TWOSEX-MSChart: The key tool for life table research and education. Entomol. Gen..

[25] Chi H., Kavousi A., Gharekhani G., Atlihan R., Özgökçe M.S., Güncan A., Gökçe A., Smith C.L., Benelli G., Guedes R.N.C. (2023). Advances in theory, data analysis, and application of the age-stage, two-sex life table for demographic research, biological control, and pest management. Entomol. Gen..

[26] Huang Y.B., Chi H. (2013). Life tables of Bactrocera cucurbitae (Diptera: Tephritidae): With an invalidation of the jackknife technique. J. Appl. Entomol..

[27] Markus M.T., Groenen P.J.F. (1998). An introduction to the bootstrap. Psychometrika.

[28] Akca I., Ayvaz T., Yazici E., Smith C.L., Chi H. (2015). Demography and Population Projection of Aphis fabae (Hemiptera: Aphididae): With Additional Comments on Life Table Research Criteria. J. Econ. Entomol..

[29] Akkopru E.P., Atlihan R., Okut H., Chi H. (2015). Demographic Assessment of Plant Cultivar Resistance to Insect Pests: A Case Study of the Dusky-Veined Walnut Aphid (Hemiptera: Callaphididae) on Five Walnut Cultivars. J. Econ. Entomol..

[30] Smucker M.D., Allan J., Carterette B. A Comparison of Statistical Significance Tests for Information Retrieval Evaluation. Proceedings of the Sixteenth ACM Conference on Information and Knowledge Management, CIKM 2007.

[31] Wei M.F., Chi H., Guo Y.F., Li X.W., Zhao L.L., Ma R.Y. (2020). Demography of *Cacopsylla chinensis* (Hemiptera: Psyllidae) Reared on Four Cultivars of *Pyrus bretschneideri* (Rosales: Rosaceae) and *P. communis* Pears With Estimations of Confidence Intervals of Specific Life Table Statistics. J. Econ. Entomol..

[32] Chi H. (1990). Timing of control based on the stage structure of pest populations: A simulation approach. J. Econ. Entomol..

[33] Chi H. TIMING-MSChart-Exe.rar. http://140.120.197.173/Ecology/prod02.htm.

[34] Dietzl G., Chen D., Schnorrer F., Su K.C., Barinova Y., Fellner M., Gasser B., Kinsey K., Oppel S., Scheiblauer S. (2007). A genome-wide transgenic RNAi library for conditional gene inactivation in Drosophila. Nature.

[35] Dubelman S., Fischer J., Zapata F., Huizinga K., Jiang C.J., Uffman J., Levine S., Carson D. (2014). Environmental Fate of Double-Stranded RNA in Agricultural Soils. PLoS ONE.

[36] Tan J.G., Levine S.L., Bachman P.M., Jensen P.D., Mueller G.M., Uffman J.P., Meng C., Song Z.H., Richards K.B., Beevers M.H. (2016). No impact of DvSnf7 RNA on honey bee (*Apis mellifera* L.) adults and larvae in dietary feeding tests. Environ. Toxicol. Chem..

[37] Song H.F., Zhang J.Q., Li D.Q., Cooper A.M.W., Silver K., Li T., Liu X.J., Ma E.B., Zhu K.Y., Zhang J.Z. (2017). A double-stranded RNA degrading enzyme reduces the efficiency of oral RNA interference in migratory locust. Insect Biochem. Mol. Biol..

[38] Gendron C.M., Pletcher S.D. (2017). MicroRNAs mir-184 and let-7 alter Drosophila metabolism and longevity. Aging Cell.

[39] Li Y., Song W., Li H., Liu C. (2021). Effects of Sublethal Concentrations of Two Pesticides on the Population Density of *Sitobion Miscanthi*. Plant Prot..

[40] Yan S., Qian J., Cai C., Ma Z., Li J., Yin M., Ren B., Shen J. (2020). Spray method application of transdermal dsRNA delivery system for efficient gene silencing and pest control on soybean aphid Aphis glycines. J. Pest Sci..

[41] Yu X.D., Killiny N. (2018). RNA interference of two glutathione S-transferase genes, *Diaphorina citri DcGSTe2* and *DcGSTd1*, increases the susceptibility of Asian citrus psyllid (Hemiptera: Liviidae) to the pesticides fenpropathrin and thiamethoxam. Pest Manag. Sci..

[42] Petrick J.S., Brower-Toland B., Jackson A.L., Kier L.D. (2013). Safety assessment of food and feed from biotechnology-derived crops employing RNA-mediated gene regulation to achieve desired traits: A scientific review. Regul. Toxicol. Pharmacol..

