# Supplementary material for: Utilizing Star Polycation Nanocarrier for the Delivery of miR-184 Agomir and Its Impact on the Life History Traits of the English Grain Aphid, Sitobion avenae
Source: Insects. 2024 Jun 19;15(6):459. doi: 10.3390/insects15060459 (PMC11203962; doi:10.3390/insects15060459)
Supplement: Supplementary file 1 [file insects-15-00459-s001.zip › insects-3045309-supplementary.pdf]

Supplementary Materials:

Figures:

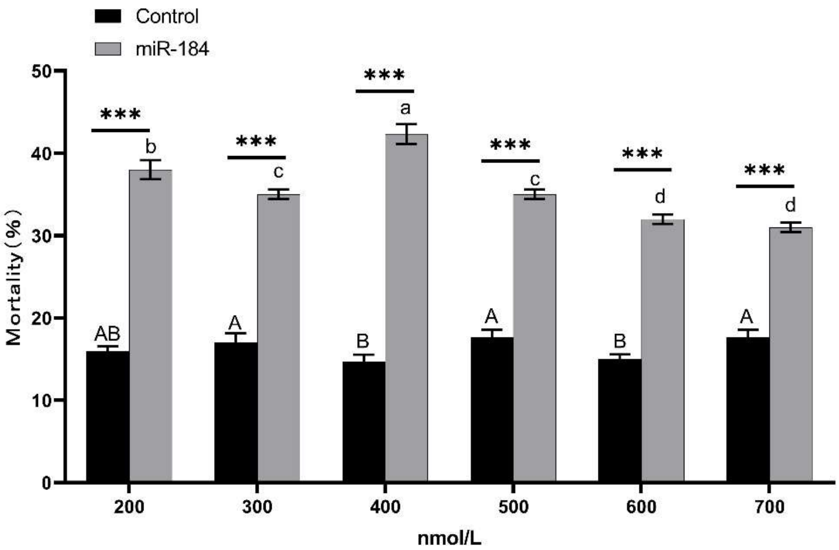

Figure S1. The impact of different concentrations of miR-184 agomir on the mortality of *S. avenae*. Control: NC agomir + nanomaterial SPc + adjuvant; miR-184: miR-184 agomir + nanomaterial SPc + adjuvant. ***: $P<0.001$. Different letters indicate significant differences ($P<0.05$, paired bootstrap test).

Tables:

Table S1. Sequence information of miRNA agomir

Name	Sequences (5′-3′)
miRNA-184 agomir	UGGACGGAGAACUGAUAAGGGCUU<br>GCCCUUAUCAGUUCUCCGUCCAUU
NC agomir	UUCUCCGAACGUGUCACGUTT<br>ACGUGACACGUUCGGAGAAT

Table S2. *S. avenae* survival rate* after miR-184 agomir treatment

Life Stage	miR-184 agomir	NC SPc	NC Water
1st instar nymph	0.93±0.02a	0.94±0.02a	0.97±0.01a
2nd instar nymph	0.66±0.04b	0.91±0.02a	0.96±0.02a
3rd instar nymph	0.88±0.03b	0.98±0.01a	0.98±0.01a
4th instar nymph	0.99±0.01a	0.99±0.01a	0.98±0.01a
Female	0.53±0.04b	0.84±0.03a	0.89±0.03a

″*″: Data are represented as mean ± SE. Different letters in the same row indicate significant differences (P<0.05, paired bootstrap test).
